# Integrated approach of guided honest disclosure and palliative care for advanced lung cancer: impacts on emotional distress, symptom burden, and patient satisfaction

**DOI:** 10.3389/fpubh.2026.1865705

**Published:** 2026-07-08

**Authors:** Huawei Huo, Jieli Wei, Xiaofang Zhang, Yingfei Li, Xinhai Zhu, Yuyao Liu

**Affiliations:** Department of Oncology, The First Affiliated Hospital of Jinan University, Guangzhou, China

**Keywords:** advanced lung cancer, emotional improvement, guided open communication, palliative care, quality of life

## Abstract

**Objective:**

To explore the feasibility and clinical value of a comprehensive nursing model that integrates guided open communication strategies with the philosophy of palliative care in patients with advanced lung cancer, and to evaluate its impact on patients’ negative emotions, pain response, cancer-related fatigue, quality of life, and nursing satisfaction.

**Methods:**

A retrospective analysis was conducted on the clinical data of 276 patients with advanced lung cancer who received treatment in our hospital from April 2023 to June 2025. According to the different nursing protocols, patients were divided into two groups: the control group (*n* = 138) received routine nursing care, while the observation group (*n* = 138) received additional comprehensive measures combining guided open communication intervention with palliative care on top of routine nursing. The two groups were compared in terms of negative emotions [Depression-Anxiety-Stress Scale (DASS-21)], cancer pain response [American Pain Society Patient Outcome Questionnaire Revised Version (APS-POQ-Modified)], cancer-related fatigue [Piper Fatigue Scale (PFS)], quality of life [European Organization for Research and Treatment of Cancer Quality of Life Questionnaire (EORTC QLQ-C30)], and nursing satisfaction [Newcastle Satisfaction with Nursing Scale (NSNS)].

**Results:**

After the intervention, anxiety score, depression score, stress score, pain intensity score, pain interference with daily life score, pain belief score, physical sensation fatigue score, affective fatigue score, behavioral fatigue score, and cognitive fatigue score in both groups decreased compared with pre-intervention; pain control satisfaction score, physical functioning score, role functioning score, cognitive functioning score, emotional functioning score, social functioning score, and global health status score increased compared with pre-intervention, with the magnitude of improvement being greater in the observation group than in the control group (*p* < 0.05). The overall nursing satisfaction in the observation group (94.20%) was higher than that in the control group (80.43%) (*p* < 0.05).

**Conclusion:**

The integration of guided open communication and palliative care in the nursing of patients with advanced lung cancer can exert a synergistic effect, not only effectively alleviating anxiety, depression, and other negative emotions, but also reducing pain and cancer-related fatigue, improving quality of life, and enhancing nursing satisfaction, with high value for clinical promotion.

## Introduction

Lung cancer is one of the malignant tumors with consistently high incidence and mortality worldwide, characterized by insidious onset, rapid progression, strong metastatic tendency, and poor prognosis (1). According to global cancer statistics, lung cancer accounts for approximately 18–20% of all cancer-related deaths, with more than two-thirds of patients diagnosed at a locally advanced or metastatic stage, losing the opportunity for radical surgery (2). In China, the number of patients with advanced lung cancer is substantial, and under the dual influence of disease progression and treatment-related side effects, their physical function, psychological status, and social adaptability generally decline, often accompanied by pain, fatigue, dyspnea, and other symptoms, severely impairing quality of life (3, 4). In addition to physical symptoms, advanced lung cancer patients also face significant psychosocial distress. Studies (5) have shown that negative emotions such as anxiety, depression, helplessness, fear, and despair are highly prevalent in this population and are positively correlated with symptoms such as pain and cancer-related fatigue. Negative emotions not only affect patients’ adherence to treatment but also further deteriorate immune function and shorten survival (6). However, in clinical nursing practice, routine care often focuses on basic treatment support and symptom relief, with insufficient systematic intervention for patients’ psychological needs and spiritual comfort, leading to inadequate psychological support and humanistic care for some patients in the terminal stage.

Guided open communication intervention is a nursing strategy emphasizing honest, open, and guided communication between healthcare providers, patients, and their families, aiming to help patients express their inner feelings more authentically, understand disease progression and the treatment process, and thereby reduce psychological stress and enhance coping ability through active listening, emotional validation, problem clarification, and positive guidance (7, 8). Palliative care, on the other hand, is a comprehensive care model centered on patient comfort and dignity, emphasizing symptom control, psychological support, social assistance, and spiritual comfort, and improving end-of-life quality of life through interdisciplinary collaboration (9). Existing studies have shown that guided open communication can effectively improve psychological adaptation in cancer patients (10), while palliative care has unique advantages in pain relief, symptom control, and quality-of-life improvement (11). However, clinical studies that organically combine the two approaches for advanced lung cancer patients remain limited. Based on this, the present study aimed to integrate guided open communication with palliative care concepts to form a comprehensive nursing model for advanced lung cancer patients and to evaluate, through retrospective analysis, its effectiveness in improving negative emotions, alleviating cancer pain, reducing cancer-related fatigue, enhancing quality of life, and increasing nursing satisfaction. The goal is to provide evidence-based support for optimizing nursing programs for advanced lung cancer patients and to offer a reference for promoting a humanistic care-oriented end-of-life care model.

## Materials and methods

### Study design and ethics

This study was a single-center, retrospective controlled study, following the STROBE reporting guidelines. The study period ranged from April 2023 to June 2025, and data were obtained from the electronic medical record system and nursing information system of the Oncology Department/Palliative Care Unit and related outpatient clinics in our hospital. As this was an analysis of previously collected data, the ethics committee approved the study and waived the requirement for renewed informed consent (Ethics Approval No.: KY-2025-260). All data were de-identified before extraction, strictly adhering to privacy and data security requirements, and were accessible only to authorized members of the research team.

### Study subjects

A total of 276 patients with advanced lung cancer who were diagnosed and received treatment/care at our hospital during the study period were consecutively enrolled. The diagnostic criteria included histopathological or cytological confirmation of non-small cell lung cancer or small cell lung cancer; for patients without available tissue evidence, enrollment was allowed if multidisciplinary team (MDT) evaluation of imaging and clinical data reached a high level of agreement. Patients were grouped according to the actual nursing plan received: Control group (*n* = 138): routine nursing care. Observation group (*n* = 138): comprehensive “Guided Honest Disclosure + Palliative Care” intervention in addition to routine nursing care. Inclusion criteria: (1) Age ≥18 years, no gender restriction; (2) Unresectable locally advanced or metastatic lung cancer; (3) ECOG performance status score 0–3 points (12); (4) Ability to complete questionnaires and communicate in Mandarin; (5) Completion of at least one baseline and one post-intervention assessment during hospitalization or outpatient follow-up; (6) Complete medical records with no more than 10% missing data for key variables. Exclusion criteria: (1) Severe psychiatric disorders or confirmed cognitive impairment affecting questionnaire completion; (2) Concurrent diagnosis of another primary malignant tumor; (3) Already under continuous deep sedation or with an expected survival of less than 8 weeks at enrollment; (4) Receipt of other structured psychological interventions within the past month; (5) Refusal or inability to cooperate with assessment; (6) Missing key outcome indicators with no possibility of data recovery. Regarding sample size, this study included the full sample available within the defined time window and did not conduct *a priori* sample size estimation.

### Nursing intervention

Grouping was based on the actual nursing pathway received, without random allocation.

### Control group

Patients in the control group received routine nursing interventions, including disease and treatment education, basic vital signs and symptom monitoring, medication and adverse reaction guidance, nutrition and activity advice, discharge guidance, routine psychological comfort, and necessary referrals for social support. Pain management followed the department’s standard procedures, with the responsible nurse assessing as needed and reporting to the physician for plan adjustment.

### Observation group

Patients in the observation group received a structured comprehensive intervention combining Guided Honest Disclosure and Palliative Care, implemented by an MDT. Key elements and workflow were as follows: (1) Team and workflow: Composition: oncologist, palliative care physician, specialist nurse (including pain management nurse), psychological counselor/social worker, dietitian, rehabilitation therapist. Workflow: complete baseline assessment and develop an individualized “care prescription” within 48 h of enrollment; hold weekly MDT meetings for dynamic optimization; keep records for all conversations and follow-ups. (2) Guided Honest Disclosure: ① Assessment and goal setting: use DASS-21 and a simplified communication needs scale at baseline to identify primary psychological distress and information needs; jointly set staged goals with patients/families (e.g., “improved sleep,” “pain under control,” “stable emotions”). ② Communication framework and techniques: adopt structured disclosure and empathy techniques (e.g., SPIKES six-step protocol (13), NURSE empathy framework (14)); follow the principle of “honest—appropriate—tolerable,” explaining illness, treatment, and prognosis in a graded manner, avoiding evasion or overcommitment. ③ Emotion identification and regulation: guide patients to name emotions and identify triggers; teach breathing relaxation, progressive muscle relaxation, and brief mindfulness exercises (10–15 min daily); provide cognitive restructuring and self-dialogue cards to address catastrophic thinking. ④ Family and caregiver involvement: hold at least one joint family meeting to unify care information, assign caregiving roles, and reduce communication conflicts and information asymmetry. ⑤ Frequency and duration: at least 2–3 structured sessions per week (40–60 min each) for 4 weeks; patients hospitalized for less than 4 weeks were followed up via phone/video to ensure total intervention duration. (3) Palliative care module: ① Comprehensive symptom management: follow the “WHO three-step analgesic ladder” (15) combined with breakthrough pain protocols, dynamically monitor using APS-POQ-Modified; standardize opioid use and prevent side effects (e.g., constipation, nausea, drowsiness) with rescue plans in place. ② Fatigue and sleep: based on PFS results, implement energy conservation strategies, daily scheduling, and individualized light aerobic plus resistance exercise programs (priority for ECOG 0–2); provide sleep hygiene education. ③ Breathing and positioning: breathing exercises (pursed-lip breathing, diaphragmatic breathing), postural drainage, oxygen therapy evaluation, and safety education. ④ Nutrition and symptom support: nutrition screening and prescriptions (energy/protein goals), oral care, symptom-specific management for nausea/loss of appetite. ⑤ Psychological and spiritual support: respect cultural and faith backgrounds, conduct discussions on the meaning of life and value clarification, refer for spiritual care as needed. ⑥ Advance Care Planning (ACP): discuss preferences and end-of-life care wishes in stages according to patient willingness, documenting treatment preferences to reduce decision burden. (4) Intervention fidelity and quality control: develop an “intervention execution checklist,” recording session topics, duration, techniques, and goal achievement; senior nurses or psychologists randomly audit ≥10% of cases weekly, providing supervision if execution rate <80%. Both groups were assessed after 4 weeks of continuous intervention.

### Observation indicators

#### Negative emotional status

Assessed before and after intervention using the Depression-Anxiety-Stress Scale (DASS-21, Cronbach’s *α* = 0.887, validity = 0.786) (16), covering anxiety, depression, and stress domains (21 items, 4-point Likert scale, scores 7–28 per domain; higher scores indicate more severe negative emotions).

#### Cancer pain response

Assessed before and after intervention using the American Pain Society Patient Outcome Questionnaire-Modified (APS-POQ-Modified, Cronbach’s *α* = 0.863, validity = 0.825) (17), including: pain intensity (3 items, 0–30 points), pain interference with activities (5 items, 0–30 points), pain beliefs (7 items, 0–35 points; higher scores indicate worse beliefs), and pain control satisfaction (3 items, 0–18 points; higher scores indicate greater satisfaction).

#### Cancer-related fatigue

Assessed before and after intervention using the Piper Fatigue Scale (PFS, Cronbach’s *α* = 0.826, validity = 0.799) (18), covering four dimensions—somatic sensory fatigue, affective fatigue, behavioral fatigue, and cognitive fatigue (22 items, each scored 0–10; higher scores indicate more severe fatigue).

#### Quality of life

Assessed before and after intervention using the EORTC QLQ-C30 (Cronbach’s *α* = 0.784, validity = 0.805) (19), including physical, role, cognitive, emotional, and social functioning plus global health status (all scored 0–100; higher scores indicate better quality of life).

#### Nursing satisfaction

Assessed after intervention using the Newcastle Satisfaction with Nursing Scales (NSNS, Cronbach’s *α* = 0.821, validity = 0.795) (20), containing 19 items rated on a 5-point Likert scale, with higher scores indicating greater satisfaction. Categories: very satisfied (≥76 points), satisfied (57–75 points), average (38–56 points), dissatisfied (<38 points). Overall satisfaction = (very satisfied + satisfied cases)/total cases ×100%.

### Statistical analysis

GraphPad Prism 8 was used for data visualization, and SPSS 25.0 was used for statistical analysis. Continuous variables were tested for normality using the Shapiro–Wilk test and expressed as mean ± standard deviation. Within-group pre- and post-intervention comparisons were performed using paired *t*-tests, while between-group comparisons were conducted using independent-samples *t*-tests. Categorical variables were expressed as counts (*n*) and percentages (%), and comparisons were performed using *χ*^2^ tests or Fisher’s exact test, as appropriate. Considering the retrospective and non-randomized nature of the present study, multivariable regression analyses were additionally performed to further evaluate the independent association between the integrated intervention model and major clinical outcomes after adjustment for potential confounding variables. Based on previous literature and clinical relevance, age, sex, pathological type, ECOG performance status, disease course, and baseline pain grade were included as covariates. Multiple linear regression analyses were used for continuous outcomes, including DASS-21, APS-POQ-Modified, PFS, and EORTC QLQ-C30 scores, while logistic regression analysis was used for overall nursing satisfaction. A theoretical causal framework based on directed acyclic graph (DAG) principles was adopted to identify clinically relevant confounding variables before model construction. Due to the observational nature of the study, residual confounding caused by unmeasured variables could not be completely excluded. All tests were two-sided with a significance level of *α* = 0.05, and *p* < 0.05 was considered statistically significant.

## Results

### Comparison of baseline characteristics between the two groups

There were no statistically significant differences between the two groups in clinical characteristics such as gender, age, pathological type, ECOG score, disease course, and baseline pain grade (*p* > 0.05), suggesting no statistically significant differences in measured baseline characteristics between the two groups, see Table 1.

**Table 1:** Comparison of baseline general data between the two groups (*x̄* ± *s*, *n* [%]).

Characteristic	Control (*n* = 138)	Observation (*n* = 138)	*t/x* ^2^	*p*
Gender	–	–	0.137	0.710
Male	86 (62.32%)	83 (60. 14%)	–	–
Female	52 (37.68%)	55 (39.86%)	–	–
Age (years)	64. 15 ± 8.42	63.84 ± 8.36	0.306	0.759
Pathological type	–	–	0.189	0.663
Non-small cell lung cancer	106 (76.81%)	109 (78.99%)	–	–
Small cell lung cancer	32 (23. 19%)	29 (21.01%)	–	–
ECOG score (points)	–	–	0.061	0.804
0–1 points	54 (39. 13%)	52 (37.68%)	–	–
2 points	57 (41.30%)	59 (42.75%)	–	–
3 points	27 (19.57%)	27 (19.57%)	–	–
Disease course (months)	11.04 ± 3.57	10.59 ± 3.34	1.081	0.280
Baseline pain grade	–	–	0.069	0.792
Mild	42 (30.43%)	40 (28.99%)	–	–
Moderate	71 (51.45%)	72 (52. 17%)	–	–
Severe	25 (18. 12%)	26 (18.84%)	–	–

### Comparison of negative emotional status between the two groups

After the intervention, anxiety scores, depression scores, and stress scores in both groups were lower than before the intervention, with a greater magnitude of change in the observation group compared with the control group (*p* < 0.05), see Table 2.

**Table 2:** Comparison of negative emotional status between the two groups (*x̄* ± *s*, points).

Outcome	Control (*n* = 138)	Observation (*n* = 138)	*t*	*p*
Anxiety score	–	–	–	–
Before intervention	19.82 ± 3.12	19.77 ± 3.05	0.134	0.893
After intervention	15.46 ± 2.85^#^	13.21 ± 2.36^#^	7.143	<0.001
Depression score	–	–	–	–
Before intervention	20.04 ± 3.24	20. 18 ± 3.31	0.355	0.722
After intervention	16.25 ± 2.91^#^	13.84 ± 2.48^#^	7.404	<0.001
Stress score	–	–	–	–
Before intervention	21.33 ± 3.10	21.41 ± 3.15	0.212	0.831
After intervention	17. 14 ± 2.77^#^	14.45 ± 2.21^#^	8.917	<0.001

Compared with before intervention in the same group, #*p* < 0.05.

### Comparison of cancer pain response between the two groups

**Figure 1:**
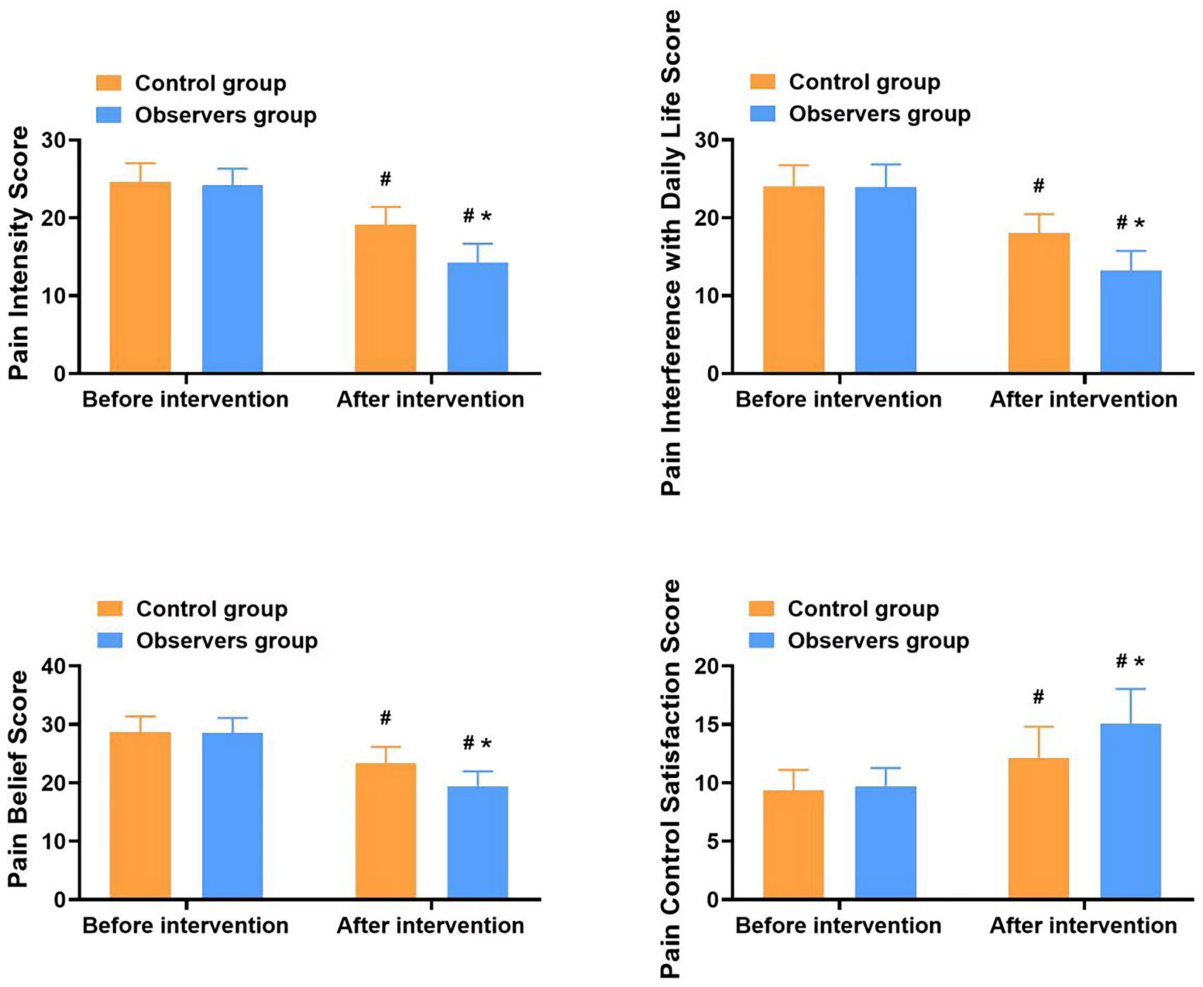
Comparison of cancer pain response between the two groups (*x̄* ± *s*, points). Compared with before intervention in the same group, ^#^*p* < 0.05; between-group comparison, ^*^p < 0.05.

After the intervention, pain intensity scores, pain interference with life scores, and pain belief scores in both groups were lower than before the intervention, while pain control satisfaction scores were higher than before the intervention; the magnitude of change was greater in the observation group compared with the control group (*p* < 0.05), see Figure 1.

### Comparison of cancer-related fatigue between the two groups

**Figure 2:**
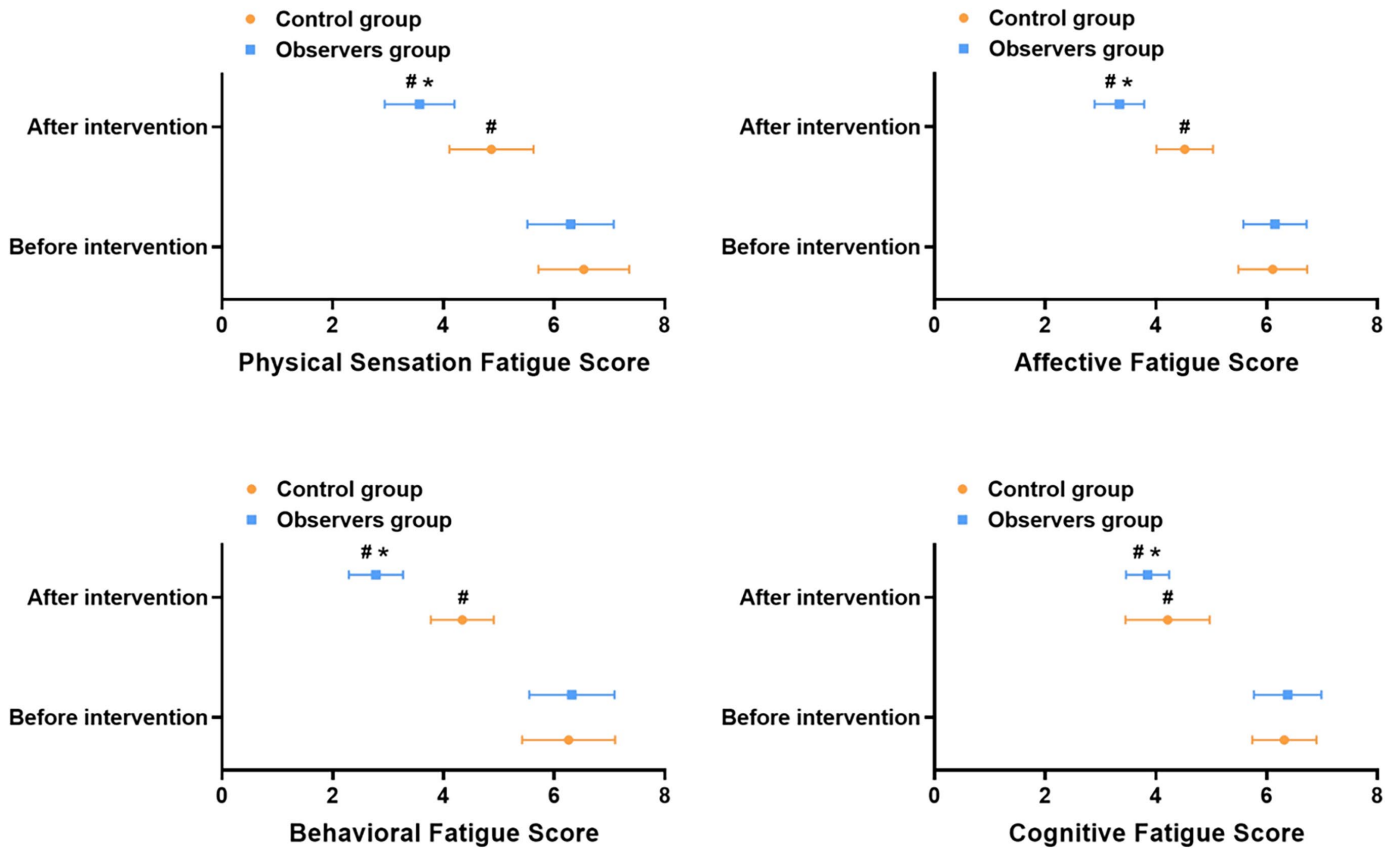
Comparison of cancer-related fatigue between the two groups (*x̄* ± *s*, points). Compared with before intervention in the same group, ^#^*p* < 0.05; between-group comparison, ^*^p < 0.05.

After the intervention, somatic sensory fatigue scores, affective fatigue scores, behavioral fatigue scores, and cognitive fatigue scores in both groups were lower than before the intervention, with a greater magnitude of change in the observation group compared with the control group (*p* < 0.05), see Figure 2.

### Comparison of quality of life between the two groups

**Figure 3:**
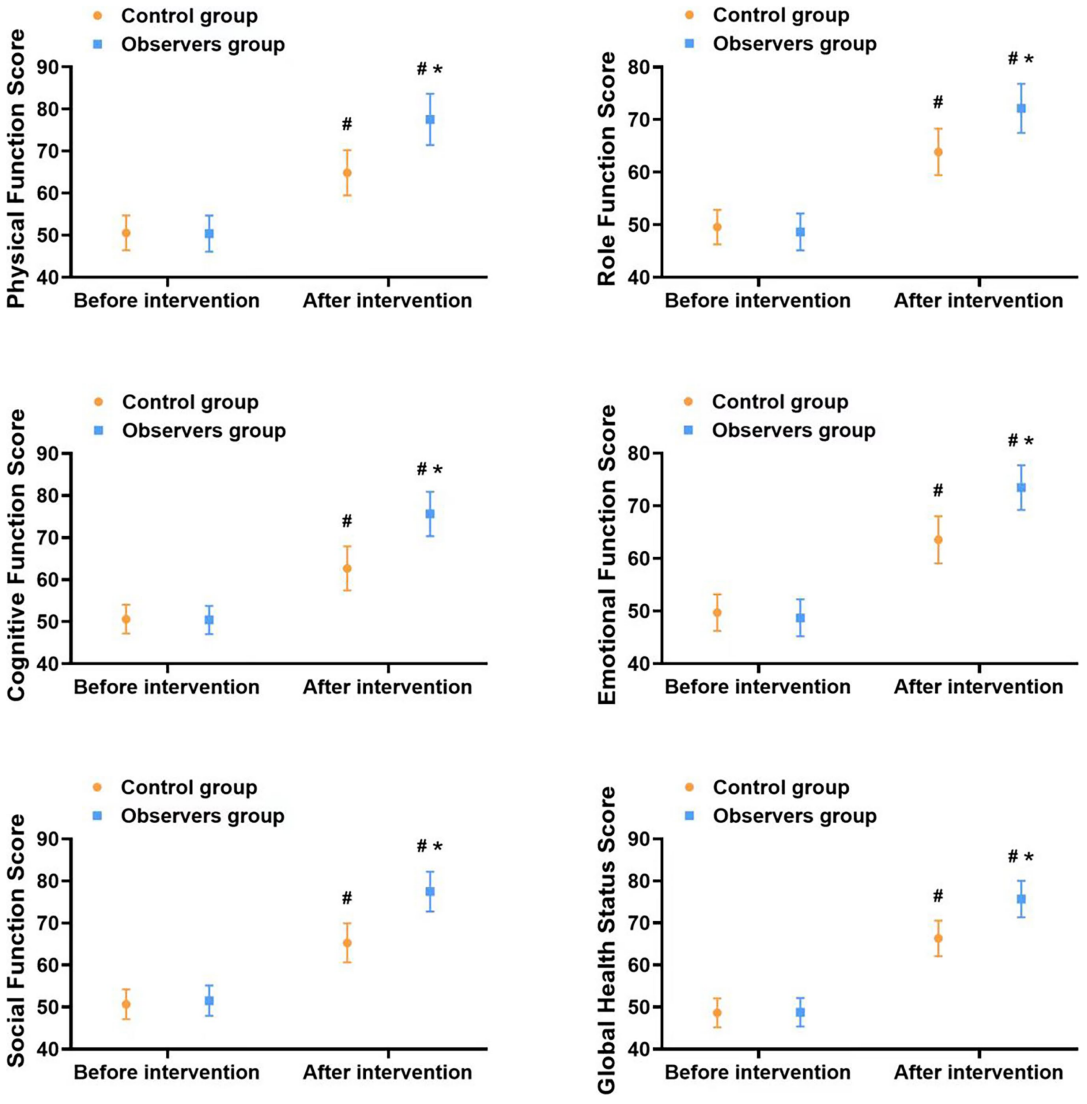
Comparison of quality of life between the two groups (*x̄* ± *s*, points). Compared with before intervention in the same group, ^#^*p* < 0.05; between-group comparison, ^*^p < 0.05.

After the intervention, physical functioning scores, role functioning scores, cognitive functioning scores, emotional functioning scores, social functioning scores, and global health status scores in both groups were higher than before the intervention, with a greater magnitude of change in the observation group compared with the control group (*p* < 0.05), see Figure 3.

### Comparison of nursing satisfaction between the two groups

The total nursing satisfaction in the observation group (94.20%) was higher than that in the control group (80.43%) (*p* < 0.05), see Table 3.

**Table 3:** Comparison of nursing satisfaction between the two groups [*n*(%)].

Satisfaction level	Control (*n* = 138)	Observation (*n* = 138)	*x* ^2^	*p*
Very satisfied	49 (35.51%)	71 (51.45%)	–	–
Satisfied	62 (44.93%)	59 (42.75%)	–	–
Average	17 (12.32%)	6 (4.35%)	–	–
Dissatisfied	10(7.25%)	2(1.45%)	–	–
Total satisfaction	111 (80.43%)	130 (94.20%)	11.812	<0.001

### Multivariable regression analysis

After adjustment for potential confounding variables, including age, sex, pathological type, ECOG performance status, disease course, and baseline pain grade, the integrated intervention model combining guided honest disclosure and palliative care remained independently associated with significantly lower anxiety, depression, stress, pain intensity, pain interference, pain belief, and cancer-related fatigue scores, as well as significantly higher pain control satisfaction, quality-of-life domain scores, and overall nursing satisfaction (all *p* < 0.05). Emotional functioning, global health status, and nursing satisfaction demonstrated relatively greater adjusted effect sizes. These findings suggest that the observed benefits were not solely explained by measured baseline clinical differences between the two groups, although residual confounding from unmeasured variables could not be completely excluded. See Table 4.

**Table 4:** Multivariable regression analysis of major clinical outcomes after adjustment for confounding variables.

Outcome variable	Regression coefficient (*β*)/OR	Standard error (SE)	95% CI	*t*/*Z* value	*p* value
Anxiety score	−2.137	0.418	−2.960 to −1.314	−5.112	<0.001
Depression score	−2.284	0.436	−3.142 to −1.426	−5.239	<0.001
Stress score	−2.517	0.451	−3.405 to −1.629	−5.581	<0.001
Pain intensity score	−1.846	0.392	−2.618 to −1.074	−4.709	<0.001
Pain interference score	−2.105	0.447	−2.985 to −1.225	−4.709	<0.001
Pain belief score	−2.436	0.508	−3.436 to −1.436	−4.795	<0.001
Pain control satisfaction score	2.184	0.473	1.253 to 3.115	4.618	<0.001
Physical sensation fatigue score	−1.965	0.431	−2.814 to −1.116	−4.559	<0.001
Affective fatigue score	−1.843	0.409	−2.649 to −1.037	−4.506	<0.001
Behavioral fatigue score	−2.021	0.438	−2.883 to −1.159	−4.614	<0.001
Cognitive fatigue score	−1.912	0.421	−2.741 to −1.083	−4.542	<0.001
Physical functioning score	8.462	1.735	5.046 to 11.878	4.877	<0.001
Role functioning score	7.984	1.628	4.779 to 11.189	4.904	<0.001
Cognitive functioning score	9.127	1.846	5.491 to 12.763	4.945	<0.001
Emotional functioning score	10.284	1.952	6.440 to 14.128	5.268	<0.001
Social functioning score	9.563	1.884	5.854 to 13.272	5.076	<0.001
Global health status score	8.915	1.726	5.518 to 12.312	5.165	<0.001
Overall nursing satisfaction	OR = 2.874	0.362	1.416 to 5.835	2.931	0.003

Multivariable regression analyses were adjusted for age, sex, pathological type, ECOG performance status, disease course, and baseline pain grade. Linear regression models were used for continuous outcomes, and logistic regression analysis was used for overall nursing satisfaction.

## Discussion

In terms of negative emotional status, this study showed that, on the basis of routine nursing, the introduction of a comprehensive nursing model integrating “guided honesty” and “palliative care” can significantly reduce depression, anxiety, and stress levels in advanced lung cancer patients within a short term (4 weeks). The possible mechanism may be that guided honesty intervention, through structured communication models (such as the SPIKES and NURSE frameworks), helps patients obtain complete and understandable disease information, thereby reducing anticipatory anxiety caused by uncertainty of information, and promotes emotional expression and regulation through empathetic responses. In addition, palliative care emphasizes symptom control and holistic care in the psychological–social–spiritual dimensions, which can reduce physical and mental burden and restore psychological security. Multiple studies (21, 22) have confirmed that tumor patients’ negative emotions are highly correlated with the ambiguity of disease-related information, and that early implementation of truthful, stratified, and gentle communication can effectively alleviate emotional distress. The results of this study are consistent with previous evidence, further confirming that integrating communication interventions with palliative concepts into a standardized nursing framework can achieve more significant short-term emotional improvements, suggesting that the synergistic effect of the two is superior to single interventions. Regarding pain response and improvement in pain management, the results showed that the observation group outperformed the control group in multiple indicators, including pain intensity, the impact of pain on daily life, and pain beliefs, while satisfaction with pain control increased significantly. This may be attributed to two aspects: first, palliative care provides systematic and individualized pharmacological and non-pharmacological pain relief programs, including the WHO three-step analgesic ladder strategy and adjuvant measures such as relaxation training and mindfulness meditation, which can reduce central sensitization and pain amplification effects; second, guided honesty communication improves patients’ understanding of pain mechanisms and pain relief programs, reduces fear and non-compliance regarding opioids and other medications, thereby improving adherence to analgesic strategies. Previous literature (23) has reported that a considerable proportion of cancer pain management failures are related to patients’ cognitive biases and insufficient communication. Compared with studies that only optimized pharmacological treatment (24), the degree of pain relief and satisfaction improvement in this study was greater, suggesting that multidimensional interventions not only improve the physiological pain process but also change patients’ pain perception and coping patterns.

In terms of relieving cancer-related fatigue, the observation group showed significant decreases in all four dimensions of the PFS scale—somatic sensory fatigue, affective fatigue, behavioral fatigue, and cognitive fatigue—indicating that the intervention alleviated cancer-related fatigue in a multidimensional manner. The mechanism may be related to multiple factors such as suppression of inflammatory responses, improvement of sleep quality, maintenance of activity levels, and emotional buffering. The individualized activity prescriptions and energy conservation strategies integrated in palliative care can prevent deconditioning, while mindfulness and breathing training can improve sleep structure and daytime fatigue; guided honesty communication can partially relieve catastrophic expectations about the disease, reducing the additive effects of psychological fatigue. Multiple randomized controlled trials (25–27) have supported the significant improvement of cancer-related fatigue by multidimensional interventions, but most studies have only focused on physiological interventions. The advantage of this study lies in the inclusion of both psychological and physiological interventions in a single implementation pathway, ensuring intervention dosage and consistency, thereby achieving a uniform decrease across all four dimensions of the PFS scale. In terms of quality of life improvement, the EORTC QLQ-C30 results showed that the observation group significantly improved in all five functional domains—physical, role, emotional, cognitive, and social functions—indicating that the comprehensive intervention brought about an overall enhancement in quality of life. This effect can be seen as a comprehensive result of the aforementioned emotional relief, pain control, and fatigue improvement. One of the cores of palliative care is integrated symptom management, which involves simultaneous interventions for pain, dyspnea, sleep disorders, and other symptoms, thereby reducing overall functional decline caused by uncontrolled single symptoms. Guided honesty communication promotes joint decision-making between patients and their families, aligning treatment goals with life goals, reducing frustration and social role conflicts caused by unmet treatment expectations. Previous studies (28, 29) have confirmed that early palliative interventions can improve quality of life, but this study showed that when palliative care is implemented synergistically with systematic communication, the degree and stability of functional domain improvement are superior. Finally, regarding nursing satisfaction, NSNS scores indicated that the nursing satisfaction rate in the observation group reached 94.20%, significantly higher than 80.43% in the control group. The increase in satisfaction not only reflects patients’ subjective recognition of nursing service quality but may also affect treatment adherence and long-term outcomes (30). Guided honesty communication makes patients feel respected emotionally and fully informed cognitively, while palliative care allows patients to experience tangible improvements in symptom management. The combination of the two significantly enhances patients’ trust in the nursing team. Studies (31) have pointed out that the quality of doctor–patient communication is highly positively correlated with nursing satisfaction, but this study shows that good communication alone is not enough to support high satisfaction—substantial improvements in symptoms and function must be achieved to form a positive feedback loop.

It should be noted that this study still has certain limitations, including: (1) this study was a single-center retrospective controlled study without random allocation, and therefore selection bias, information bias, and residual confounding could not be completely avoided. Although multivariable regression analyses were performed to adjust for major observed confounding factors based on theoretical causal assumptions and existing literature, unmeasured confounders may still have influenced the outcomes. (2) The intervention period was short, observing only short-term effects, without including long-term outcomes such as survival period and readmission rate, nor measuring biological mediators such as inflammatory factors and cortisol levels. (3) Differences in the communication skills and palliative care experience of implementers may affect intervention effectiveness. Therefore, future studies should adopt multicenter randomized controlled designs, extend follow-up periods, and combine biological indicators with economic evaluations to explore the intervention’s mechanism pathways, cost-effectiveness, and stratification strategies for applicable populations.

## Conclusion

The results of this study indicate that, on the basis of routine nursing, integrating guided honesty intervention and palliative care into a comprehensive nursing model can significantly alleviate negative emotions such as depression, anxiety, and stress in patients with advanced lung cancer, effectively reduce cancer pain and cancer-related fatigue, and significantly improve nursing satisfaction while enhancing patients’ quality of life in multiple dimensions. The advantage of this model lies in combining high-quality doctor–patient communication with holistic, continuous symptom management, achieving a synergistic effect of psychological support and physical intervention, thereby obtaining significant short-term clinical benefits. Given its good clinical feasibility and patient acceptance, this intervention model has high potential for promotion and application in the nursing of advanced lung cancer and even other advanced cancers. However, multicenter, large-sample, long-term follow-up randomized controlled trials are still needed in the future to further verify its long-term efficacy, mechanisms of action, and cost-effectiveness.

## Data Availability

The original contributions presented in the study are included in the article/supplementary material, further inquiries can be directed to the corresponding author.
